# Evaluating Thailand’s malaria reactive surveillance and response strategies for malaria elimination: a mixed-method study

**DOI:** 10.1186/s40249-025-01382-w

**Published:** 2025-10-30

**Authors:** Win Han Oo, Wanlapa Roobsoong, Nilar Aye Tun, Kaung Myat Khant, Win Htike, Mondha Kengganpanich, Paul A. Agius, Jetsumon Sattabongkot, Freya J. I. Fowkes

**Affiliations:** 1 Centre for Epidemiology and Biostatistics, Melbourne School of Population and Global Health, University of Melbourne, Melbourne, VIC Australia; 2 Health Security and Pandemic Preparedness Program, Burnet Institute, Melbourne, VIC Australia; 3 Mahidol Vivax Research Unit, Faculty of Tropical Medicine, Mahidol University, Bangkok, Thailand; 4 Health Security Program, Burnet Institute Myanmar, Yangon, Myanmar; 5 Faculty of Health, Deakin University, Melbourne, VIC Australia; 6 Department of Health Education and Behavioral Sciences, Faculty of Public Health, Mahidol University, Bangkok, Thailand; 7 Department of Epidemiology and Preventive Medicine, Monash University, Melbourne, VIC Australia

**Keywords:** Reactive surveillance and responses, Malaria, Elimination, Thailand

## Abstract

**Background:**

Thailand aims to eliminate malaria in all provinces by 2030, where 37 out of 77 provinces have been verified as malaria-free by 2021. Thailand is accelerating its elimination activities and preventing re-establishment of malaria through implementation of the 1-3-7 reactive surveillance and response (RASR) strategy, which entails case notification within one day, case investigation within three days and case investigation survey within seven days after detecting an index malaria case. This study aimed to assess how this 1-3-7 malaria RASR strategy was implemented to understand how it may be optimised to achieve national malaria elimination goals.

**Methods:**

A cross-sectional mixed-method study was conducted in Tak, Songkhla and Yala provinces of Thailand between January and April 2023. Quantitative survey with purposively recruited malaria programme stakeholders (*n* = 33) and frontline malaria service providers (FMSPs) (*n* = 41), qualitative focus group discussions with malaria programme stakeholders, FMSPs and mobile migrant population, and individual in-depth interviews with malaria programme stakeholders were conducted face-to-face in Thai language. Quantitative and qualitative data were analysed descriptively and thematically.

**Results:**

About half of survey participants (40/74, 54.1%) perceived that malaria cases were notified within the specified one day timeframe. Almost all participants (73/74, 98.7%) reported that case investigation, focus investigation and response activities were conducted within the required timeframes, except in border areas where malaria burden was high. Despite every participant (74/74, 100.0%) being aware of and considering the 1-3-7 strategy as efficient, challenges such as limited human resources and budget, low levels of community participation, and difficult terrain and telecommunication were reported in performing RASR activities.

**Conclusions:**

The overall implementation of RASR activities was reported to be timely except for malaria case notification and focus response activities in border areas. Despite good acceptability towards RASR activities, the 1-3-7 strategy is not a one-size-fits-all approach; its feasibility for implementation depends on health system capacity and malaria incidence of the area. There are still several challenges to be addressed particularly for successful implementation of RASR activities for cross-border malaria.

**Graphical Abstract:**

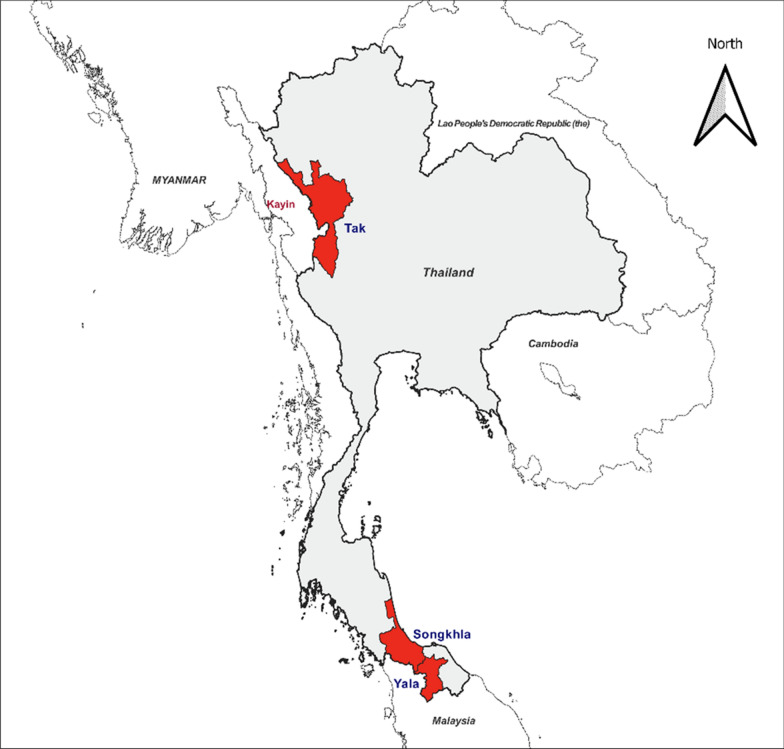

**Supplementary Information:**

The online version contains supplementary material available at 10.1186/s40249-025-01382-w.

## Background

Thailand is a Greater Mekong Subregion country and malaria endemic. The Mekong Malaria Elimination Programme verified 37 out of 77 provinces in Thailand as malaria-free in 2021 [1]. Thailand performed well in working towards malaria elimination and met the 2022 milestone of the Global Technical Strategy for Malaria Elimination—reducing malaria incidence by 55% compared with 2015 [2]. Thus, Thailand aims to eliminate malaria in all provinces by 2030 [3]. Nevertheless, with confirmed malaria cases of 3,216 in 2021 [4], 10,155 in 2022 [5] and 16,675 in 2023 [6] respectively, the malaria burden in Thailand has increased again in recent years concentrated at border areas.

To achieve malaria elimination by 2030, detection, treatment and response to every malaria case is critical. In Thailand, the National Malaria Elimination Strategy (2017–2026) [7] advises a strengthened surveillance system as the core intervention is a key component in order to accelerate malaria elimination. The disease surveillance system encompasses case detection, and epidemiological investigations of detected cases and their foci. Active case detection activities cover pro-active case detection, consisting of special case detection and mobile malaria clinics, and reactive case detection (RACD) consisting of mass blood and case investigation surveys. Passive case detection takes place at different levels of health facilities including malaria clinics, general hospitals, health promoting hospitals and malaria posts [3]. To effectively implement malaria surveillance and response activities, Thailand adopted the 1-3-7 reactive surveillance and response (RASR) strategy, which entails case notification within one day, case investigation (CI) within three days and case investigation survey within seven days following detection of an index malaria case [8].

Since Thailand is accelerating its elimination effort in endemic provinces and preventing reestablishment of malaria in eliminated provinces [9], it is crucial to effectively implement the RASR strategies so that the surveillance system can capture all malaria cases and prevent onward transmission.

To provide recommendations for improvement in surveillance system and pledge national malaria elimination as targeted, a mixed-method evaluation study was conducted. This paper describes the implementation of RASR activities, adherence to its 1-3-7 timelines and challenges encountered during the implementation. It also explores the acceptability and feasibility of the RASR strategy among the malaria programme stakeholders, frontline malaria service providers (FMSPs) and mobile and migrant populations (MMPs) in Tak, Yala and Songkhla provinces of Thailand.

## Methods

### Study design

This study is a mixed-method study that employed quantitative cross-sectional surveys, qualitative focus group discussions (FGDs) and semi-structured individual in-depth interviews (IDIs). The reporting of the study adhered to the Strengthening the Reporting of Observational Studies in Epidemiology [10] and Standards for Reporting Qualitative Research checklists [11] (Supplementary materials 1).

Using the questionnaires (Supplementary materials 2), the two surveys were carried out among malaria programme stakeholders (*n* = 33) who were responsible for managing and overseeing the RASR activities and FMSPs (*n* = 41) who performed the RASR activities (Supplementary materials 3: Additional Table 1). Subsets of survey participants (*n* = 26 malaria programme stakeholders in four FGDs and *n* = 34 FMSPs in seven FGDs) and *n* = 22 MMPs in five FGDs provided their inputs qualitatively. Three IDIs were conducted with the malaria programme stakeholders responsible for designing and overviewing RASR policies and strategies. FGD and IDI topic guides (Supplementary material 2) were used.

### Study setting

This study was conducted in three provinces of Thailand: Tak, Songkhla and Yala (Supplementary materials 3: Additional Table 2). Tak is located in the west of Thailand and neighbouring with Kayin State of Myanmar, and thus a malaria high transmission area. The annual parasite incidence (API) of Tak ranged from zero to ten per thousand per year between 2020–2024 [12] showing a complex malaria epidemiology. Songkhla and Yala are situated in the south of Thailand bordering Malaysia with a varying API of zero to five per thousand per year during the same period [8, 13].

### Sampling

Participants were recruited purposively based on their roles and experience with implementing malaria control and RASR activities in the selected provinces, and their availability to participate in the survey. Sample size was not determined due to limited availability of eligible participants and no intention for inference about the population. A subset of FMSPs and malaria programme stakeholders who participated in the surveys were intentionally invited to participate in FGDs for discussion regarding RASR implementation issues based on diversity of their roles in performing RASR activities. Military and paramilitary staff, and villagers from border areas were purposively selected as MMPs to participate in FGD regardless of citizenship and residential status as they themselves identified as forest-goers or migrants and are familiar with the MMP communities. Malaria programme stakeholders who were expert in malaria control, well-experienced with the RASR strategy, and responsible for designing and overviewing RASR policies and strategies were deliberately recruited for the IDI. For qualitative consultations, data saturation was used to cease recruitment.

### Data collection, management and analyses

Between January to April 2023, quantitative and qualitative data collections were conducted confidentially in private locations in the selected provinces. The face-to-face data collections were performed by Mahidol University Faculty of Tropical Medicine staff using the primary language of the participants, Thai. The survey, and IDI and FGD data collections lasted approximately 60 min and 1.3–2 h, respectively.

Two similar sets of survey questionnaires were constructed in English and translated into Thai in the Epicollect5 application (Supplementary materials 2). Once the data was collected via Epicollect5, they were exported to comma separated values (.csv) files, and participants’ open responses in local language were translated to English, which were cleaned, managed and analysed descriptively in the statistical software Stata version 17.0 (Stata Corp, College Station TX, USA).

The FGDs and IDIs were audio-recorded with the consent of the participants. Audio records were transcribed verbatim and translated into English. They were then organised, managed and analysed in the software NVivo version 12 (Lumivero, Denver CO, USA). Thematic (deductive followed by inductive) analysis was undertaken using the qualitative descriptive approach [14]. A deductive thematic framework that includes coding definitions, themes and subthemes were developed before the analysis. Two researchers immersed the data by reading the transcripts three times. Each of the two researchers then coded data separately, referring to the deductive thematic framework. Emerging themes were then added into the framework inductively. After coding, the researchers then discussed themes and subthemes to reach a consensus on the final thematic framework and interpretation [15]. The results were reported thematically.

## Results

### Implementation of RASR activities

#### Case notification

The majority of stakeholders responded that telephone calling and online messaging platform such as ‘LINE’ group chats were used in initial reporting of malaria cases to a focal person (31/33, 93.9% each). According to FMSPs, an online messaging platform was their main method to initially report to their supervisor or organization (30/41, 73.2%) (Table 1). The majority of FMSPs surveyed reported that there was mobile phone signal (36/41, 87.8%) and internet access (33/41, 80.5%) in the villages, and that access was good enough to do case reporting activities (33/41, 80.5%) (Supplementary material 3: Additional Table 3). However, there were still areas where the mobile phone signal was not good enough to make an immediate call (11/41, 26.8%) where paper-based reporting was used. About half of survey participants (40/74, 54.0%) assumed that more than 90% of cases were timely notified (i.e., within 24 h of diagnosis) (Table 1).

**Table 1:** Methods used in and reported timeliness of case notification after detecting an index malaria case

Variables	Overall *n* = 74 (%)	Malaria programme stakeholders *n* = 33 (%)	Frontline malaria service providers *n* = 41 (%)
Methods of case notification†			
Messaging platform (e.g. LINE App)	61 (82.4)	31 (93.9)	30 (73.2)
Telephone calling	56 (75.7)	31 (93.9)	25 (61.0)
Paper-based reporting	46 (62.2)	30 (90.9)	16 (39.0)
Online reporting system (mHealth)	41 (55.4)	27 (81.8)	14 (34.2)
Reported timeliness of case notification*			
Never	0 (0.0)	0 (0.0)	0 (0.0)
Occasionally (less than 20%)	0 (0.0)	0 (0.0)	0 (0.0)
Sometimes (20–50%)	7 (9.5)	4 (12.1)	3 (7.3)
More often than not (50–75%)	3 (4.1)	1 (3.0)	2 (4.9)
Usually (more than 75%)	24 (32.4)	13 (39.4)	11 (26.8)
Nearly always (more than 90%)	40 (54.0)	15 (45.5)	25 (61.0)

**†**Multiple responses; *Survey participants reported perceived percentages of cases notified timely within 24 h after diagnosis among all cases detected

In FGD, FMSPs reported that formal malaria case notification from hospitals did not follow the “1” part of the 1-3-7 timeframe due to different vertical management systems. Nevertheless, hospital staff used LINE group chat for informal alternative malaria case notification to district public health staff. In these scenarios, malaria programme stakeholders reminded FMSPs to take actions such as treatment follow up via LINE.

During an IDI, a malaria programme stakeholder reported that delayed sequential reporting cascade from hospitals was due to a lack of understanding of the 1-3-7 timeline by hospital staff. Additionally, the disease control team received reports late due to hospital staff reporting malaria case data to district public health offices without tending to the 1-3-7 timeframe. By the time the hospital reported the malaria cases to vector-borne diseases (VBD) unit, the patients had already received treatment and left the hospital and difficulty locating patients to perform CI caused delays in subsequent steps in the 1-3-7 timeframe.

For instance, if the hospital notifies us three days after encountering (diagnosis of malaria) a patient, then measure 1 (case notification) is not met, measure 3 (case investigation) is not met, and measure 7 (focus investigation and responses) will only get four days to complete to meet the deadline of 7 days. (Malaria programme stakeholder, FGD)

In FGDs, FMSPs reported that the mHealth application (the online application that has been used for malaria case management and reporting in Thailand) had problems with reporting malaria cases. The android devices provided by the government had low performance and did not meet the minimum requirement of the mHealth programme causing the application to be slow and unresponsive. Additionally, FMSPs of over 60 years encountered difficulties in using the application due to the low level of information technology literacy.

#### Case investigation

Case investigation in Thailand includes investigation of the source of the infection (where the index case gets malaria) and site of infection (where the index case stays). This information is used for decision making.

##### Policy and trigger

Most of the stakeholders (31/33, 93.9%) and FMSPs (38/41, 92.7%) reported there is a policy to conduct CI whether the case is indigenous or imported. Very few (1/33, 3.0% of the stakeholders and 3/41, 7.3% of FMSPs) reported that CI must be conducted for indigenous cases only (Table 2).

**Table 2:** Policy to conduct case investigation, and its reported completeness and timeliness in Thailand

Variables	Overall *n* = 74 (%)	Malaria programme stakeholders *n* = 33 (%)	Frontline malaria service providers *n* = 41 (%)
Program’s policy to conduct case investigation			
All indigenous and imported cases	69 (93.2)	31 (93.9)	38 (92.7)
Indigenous cases only	4 (5.4)	1 (3.0)	3 (7.3)
Imported cases only	0 (0.0)	0 (0.0)	0 (0.0)
Other	1 (1.4)	1 (3.0)	0 (0.0)
Reported completeness of case investigation*			
< 20%	3 (4.1)	1 (3.0)	2 (4.9)
> 20 to 50%	4 (5.4)	0 (0.0)	4 (9.8)
> 50 to 75%	5 (6.8)	1 (3.0)	4 (9.8)
> 75 to < 100%	24 (32.4)	15 (45.5)	9 (22.0)
100%	38 (51.4)	16 (48.5)	22 (53.7)
Time from positive case recording to case investigation initiated**			
Within 24 h	45 (60.8)	18 (54.6)	27 (65.9)
Within 48 h	8 (10.8)	2 (6.1)	6 (14.6)
Within 72 h	20 (27.0)	13 (39.4)	7 (17.1)
Within 1 week	1 (1.4)	0 (0.0)	1 (2.4)

*Survey participants reported perceived percentages of cases investigated among all cases detected

**Survey participants estimated time taken from a positive index malaria case recorded to initiation of case investigation

##### Procedure

The majority of malaria programme stakeholders (28/33, 84.9%) and all FMSPs (41/41, 100.0%) responded in surveys that there was a standard operating procedure for CI, which was self-reported to be followed by all surveyed FMSPs. Moreover, every stakeholder and FMSP responded there was a specific form to be used in CI. Almost all survey participants mentioned that CI involved visiting the index case (71/74, 96.0%), checking the case’s malaria preventive measures (72/74, 97.3%), and providing education about malaria risk factors and prevention (73/74, 98.7%).

Nearly two thirds (23/33, 69.7% of stakeholders and 24/41, 58.5% of FMSPs) answered that the index case was contacted via telephone in advance of the visit. Other methods of appointments were made in coordination with a village health volunteer or responsible person (malaria post) in that area, by issuing appointment card, or by providing a follow-up schedule to patients who obtained their diagnosis at a malaria clinic. When an index case was not home, stakeholders (22/33, 66.7%) and FMSPs (25/41, 61.0%) performed a second visit within the same day or the next day (Supplementary materials 3: Additional Table 4).

During CI, index case location was mapped either by various applications (e.g., Geographic Information System/Global Positioning System, mHealth, Google map) (37/74, 50.0%), or manually using a community map (17/74, 23.0%). (Supplementary materials 3: Additional Table 5) when there was no internet signal. The mHealth application was helpful in location mapping of the index case and checking the area for malaria screening. However, it was outdated and its use among FMSPs declined.

FMSPs reported collection of travel information within the district of residence (24/27, 88.9%) and other districts (19/27, 70.4%), but less than half (11/27, 40.7%) collected travel history outside Thailand, whereas almost all malaria stakeholders mentioned all three types for collection of travel information (30/30, 100%) (Supplementary materials 3: Additional Table 5). In FGDs, malaria programme stakeholders reported that malaria clinics asked for the index case’s name, address, daily activities and their travel history in the past month and verified the information by asking neighbours and friends.

Survey participants answered that the criterion that Thailand’s malaria programmes mostly use in defining imported cases (the source of infection is outside their responsible areas) is that the cases occurred within the country but from a different province/district/other administrative unit (29/33, 87.9% and 34/41, 82.9%). (Supplementary materials 3: Additional Table 6).

##### Reported completeness and timeliness

Malaria programme stakeholders and FMSPs reported achieving over 75% CI completeness (93.9% and 75.6%, respectively). All malaria stakeholders (33/33, 100.0%) and almost all FMSPs (40/41, 97.6%) stated that CI was done in a timely manner (i.e., at most within 72 h) (Table 2).

Both malaria programme stakeholders and FMSPs responded in surveys that the main reason of not achieving a 100% completeness in CI was that the malaria case could not be located (17/33, 51.5% and 19/41, 46.3%, respectively). Inaccessible location of the malaria case was the second most cited reason for incomplete CI (21/74, 28.4%). Additionally, stakeholders reported that CI could not be completed in the event that an index case crossed the border to Thailand to receive diagnosis and treatment before returning to their home country (10/33, 30.3% and 3/41, 7.3%) (Supplementary materials 3: Additional Table 7). In the FGDs, migrants from Tak Province stated that they cooperated with FMSPs regardless of migration status. A FMSP added that malaria cases from refugee camps should be reported separately because these cases could not be investigated due to access restrictions to the camps.

It's important to distinguish cases from the camps…. because you may not be able to conduct investigations there. (FMSP, Tak, FGD)

#### Focus investigation and responses

##### RACD and community malaria screening

The majority of surveyed malaria programme stakeholders responded that their programme routinely conducted foci investigations (28/33, 84.9%) and RACD (27/33, 81.8%). The majority of survey respondents reported that local infections detected through passive case detection as well as both local and imported cases can be a trigger for RACD (13/33, 39.4% each), while only a few reported there being no trigger for RACD in their programme. The majority of FMSPs, responded that every indigenous case is a trigger for RACD in their programme (30/41, 73.2%). The majority of both malaria programme stakeholders and FMSPs responded that a single confirmed case was enough to trigger RACD (20/33, 60.6% and 26/41, 63.4%, respectively), and that community screening was triggered by both local and imported cases (22/33, 67% and 30/41, 73%, respectively) (Supplementary material 3: Additional Table 8). However, FGD with MMPs revealed that RACD and screening activities were not conducted in military bases.

Investigations and blood tests for individuals within the (military) bases or accommodations are not common. (Soldier MMP, Thasongyang, FGD)

A majority of malaria programme stakeholders (30/32, 90.9% and 26/32, 78.8%) and FMSPs (36/41, 87.8% and 35/41, 85.4%) from the survey said that they always screened household members and neighbours of the index case, respectively in RACD regardless of fever. The majority of participants (66.7% of malaria programme stakeholders (*n* = 22/33) and 41.5% of FMSPs (*n* = 17/41) cited all three of the threshold criteria, including screening based on a specified number of neighbouring households (typically 10–15 households), people around (typically 50 people) or geographical radius (typically 1000 m) (Supplementary materials 3: Additional Table 9).

FGDs reported that the screening extent differed according to geographic area and frequency of occurrence of index malaria cases. FMSPs reported testing 50–100 people or 80% of people within a 100-m radius of the index case. If the households were sparse within the radius, out-of-radius testing was performed to achieve the targeted number of people to be screened. Malaria programme stakeholders from Yala reported that the number of people screened depended on the budget allowance provided by international multilateral donors for the malaria elimination program. If malaria cases are reported in a village for two to four consecutive weeks, screening for RACD might increase to up to 150 people or the entire village.

Malaria programme stakeholders highlighted that screening 50 people is not enough to yield a positive case and interrupt malaria transmission in the area as the screening of 50 people did not find any positive cases, but positive cases emerged in the area in a few days after the screening.

When we visited village A and B for 1-3-7 activities, we got 50 blood samples and finished tasks smoothly. We didn't find any positive cases. Three days later, there was a case in the area detected again. We did 1-3-7 again for the new case and also didn't find anyone positive. That means the 50-point case investigation survey hasn't hit the mark yet. (Malaria programme stakeholder, Yala, FGD)

In surveys, malaria programme stakeholders stated rapid diagnostic tests (RDT) and microscopy as diagnostic tools most frequently used when conducting RACD (30/33, 90.9% each), while FMSPs reported RDTs as the most frequently used (37/41, 90.2%), followed by microscopy (27/41, 65.9%) (Table 3).

**Table 3:** Diagnostic tools used for reactive case detection

Diagnostic tool for reactive case detection†	Overall *n* = 74 (%)	Malaria programme stakeholders *n* = 33 (%)	Frontline malaria service providers *n* = 41 (%)
Microscopy	57 (77.0)	30 (90.9)	27 (65.9)
Rapid diagnostic test	67 (90.5)	30 (90.9)	37 (90.2)
Polymerase chain reaction	6 (8.1)	5 (15.2)	1 (2.4)
Clinical diagnosis	3 (4.1)	2 (6.1)	1 (2.4)

^†^Multiple responses

Participants in the surveys reported diagnostic tools used for reactive case detection

In FGD, FMSPs reported that they tested with RDTs first, then confirmed the results with microscopy and that this approach was not without challenges. Sending the blood films for microscopy, particularly from remote areas, could result in delays in confirmation of diagnosis of up to five days, and the quality of blood films may be jeopardised due to humidity.

MPs (malaria posts) in remote areas might take two or three days to send the slides (blood films to the lab), aiming to send them altogether at once on Wednesday. This causes issues (delay in sending blood films collected earlier days of the week). (FMSP, Yala, FGD)

Additionally, it was reported that slide preparation during RACD was challenging. The malaria posts had to send blood films to the microscopist within two or three days. They prepared blood films hurriedly during the community screening. As a result, the quality of the blood film might not meet the standard quality as some might not be dried completely or might contain dust.

##### Focus responses

Well over half of survey participants stated that identification of a malaria focus triggered awareness raising of malaria transmission (48/74, 64.9%), malaria prevention (45/74, 60.8%) and additional vector control (44/74, 59.5%) activities (Table 4). FGD findings revealed that focus response activities included monitoring malaria treatment compliance, giving health education, distributing long lasting insecticide-treated mosquito nets and repellent, controlling larvae and indoor residual spraying (IRS) or fogging. FMSPs also reported that the frequency of focus response activities varied with the frequency of occurrence of malaria cases.

**Table 4:** Response activities after identifying a malaria focus

Response activities	Overall *n* = 74 (%)	Malaria programme stakeholders *n* = 33 (%)	Frontline malaria service providers *n* = 41 (%)
For everyone in the focus**†**			
Health education on transmission	48 (64.9)	22 (66.7)	26 (63.4)
Health education on prevention	45 (60.8)	23 (69.7)	22 (53.7)
Providing additional vector control measures	44 (59.5)	22 (66.7)	22 (53.7)
Entomological surveillance	32 (43.2)	18 (54.6)	14 (34.2)
Spot checking mosquito-breeding sites	12 (16.2)	9 (27.3)	3 (7.3)
Targeted for mobile and migrant populations†			
Long lasting insecticide-treated mosquito nets distribution	–	5 (15.2)	–
Targeted malaria testing*	–	4 (12.1)	–
Mass malaria screening**	–	7 (21.2)	–

^†^Multiple responses; *Targeted malaria testing upon return from worksites or forest; **Mass malaria screening among mobile and migrant populations; – Not applicable

Participants in the surveys reported focus response activities (for everyone in the focus and additional targeted interventions for mobile and migrant populations) conducted after identifying a malaria focus

Specific malaria focus response activities among MMPs or forest goers were targeted malaria testing upon return and insecticide-treated net distribution (Table 4). MMPs from Yala reported that when the migrants returned to the camp from their home, they were tested for malaria. If there were any positive cases, they were treated with medication followed by fogging, or IRS of their households. However, some MMPs from Thasongyang reported that malaria officials only performed malaria testing and treatment in their areas.

They schedule follow-up blood testing and provide medication individually. There haven’t been any malaria officials coming to the park to do anything. (MMP, Thasongyang, FGD)

##### Timeliness of focus investigation and responses

Almost all surveyed stakeholders agreed that focus investigation (32/33, 97.0%) and response (33/33, 100.0%) activities were conducted in a timely manner, that is, within seven days. However, in FGDs malaria stakeholders reported that the data in malaria information system showed delays in focus investigation and response while actual fieldworks were completed in time. The malaria information system, which is updated weekly, merged all the cases within a seven-day timeframe. It merged the cases regardless of index or secondary cases and counted them as ‘one’ for focus response activities. This leads to errors in counting the number of cases for which focus investigation and response activities were completed within seven days. This problem occurred especially in areas of high malaria incidence, resulting in cost reimbursement issues for community screening and focus response activities.

Because it's (reporting of focus investigation and response activities) cut off on Tuesday of a week, some activities were recorded as ‘delayed’ wrongly. For example, if a case pops up on Monday and you planned to response it on Wednesday, it should be within the timeframe. Nevertheless, you failed to follow the 1-3-7 timeframe as it passed Tuesday, the deadline to take action. The protocol is to cut immediately (after Tuesday) even if it's not yet reached 7 days (counting from the day of case notification). (Malaria programme stakeholders, Yala, FGD)

In such areas with high malaria incidence, the time constraint of the 1-3-7 approach limited the coverage of focus response activities. The malaria control team preferred timeliness within seven days to ensure effectiveness of the focus response activities. Sometimes, after performing focus response activities for an index case in a village, there might be another index case in a different radius but in the same village. On such occasion, focus response activities were performed only for the first index case. A malaria programme stakeholder suggested in an IDI that there would have been better completion of focus investigation and response activities if there was no pressure of the seven-day completion deadline. One malaria programme stakeholder also expressed that the 1-3-7 strategy prioritised the timeliness of focus response activities over their effectiveness.

If we consider that it is not necessary to strictly adhere to the 7-day timeline (for focus investigation), we will instead aim on accomplishing the task (focus investigation and responses) regardless of completion date. (Malaria programme stakeholder, Songkhla, IDI)

### Challenges in RASR implementation

Although many of the survey participants said there were no challenges in implementing RASR activities, several were revealed during surveys and qualitative consultations (Details in Supplementary material 3: Additional Table 10).

#### Human resources limitation

FGDs and IDIs revealed that limitation of human resources was a challenge to conduct RASR activities. When there were many cases, sufficient workforce was not available to complete the required response activities within seven days. Skilled, experienced malaria personnel were retiring and there were not replacements for them. Only a few newcomers could perform the IRS properly. With declining malaria cases in the southern provinces of Thailand, implementing partners were no longer interested in malaria control, making it difficult to recruit personnel to perform RASR activities. There was also loss of workforce due to health system changes. Primary health centres were transferred, together with their workforces, from the Ministry of Public Health to the Ministry of Interior, where the scope of work and priorities of health centres were reformed, leaving RASR activities as low priority tasks. Thus, the Office of Disease Control in the Ministry of Public Health may no longer use the workforce of primary health centres for malaria RASR activities.

Currently, we're addressing the past issues of having many patients and severe outbreaks, coupled with a shortage of staff. We have only one or two providers per malaria clinic (MC), one of them being the head. Some MCs have none (staff) at all. There’s only one (assigned) person, but they're not even stationed in that MC. (Malaria programme stakeholder, Yala, FGD)

Nevertheless, the presence of malaria posts and village health volunteers as part of the RASR workforce has been a major facilitator of the 1-3-7 strategy’s feasibility. Malaria posts in particular hold a unique advantage due to the community’s trust in their services. This trust enables them to engage more effectively with the community compared to VBD units. Malaria post workers and volunteers are responsive to malaria programme stakeholders and execute RASR activities promptly and competently, demonstrating skills such as preparing high-quality blood films. Their commitment to malaria elimination is evident, with many volunteering without financial support and maintaining strong peer coordination. According to FGDs, their dedication has fostered community recognition and engagement in malaria elimination efforts, indicating the potential for expanding their roles in RASR activities.

The good thing about our province is that staff from MPs can enter the area and make blood films. Sometimes, their slides are more beautiful than ours. We have training almost every year. We ask them to help for whatever we want. This is the advantage of having MPs. (Malaria programme stakeholders, Yala, FGD)

#### Budget limitation

During IDIs, a malaria programme stakeholder revealed that the process of budget allocation for RASR activities had restrictions. There were limitations in RACD budget. Reimbursement was only provided for the first 50 microscopic slides in RACD executed within seven days after notification, and only for one index case per village regardless of the number of malaria cases in that village. This led to incomplete screening in the community and failure to perform effective focus response. This was superimposed by an insufficient budget for vector surveys, IRS and long lasting insecticide-treated mosquito net distribution, resulting in a malaria outbreak.

You do 200 slides (malaria microscopy). But you can claim only up to 50 slides, leaving 150 slides not reimbursed. (Malaria programme stakeholder, Yala, FGD)

#### Poor community participation

One of the key challenges in timely implementation of RASR activities is poor community participation in some areas. Findings from FGDs revealed that many community members do not view malaria prevention as a priority, perceiving the disease as non-fatal and easily curable with free treatment. Additionally, the malaria patients, particularly migrants crossing from the Myanmar border, often withhold accurate information about their travel histories due to fears related to undocumented migration. It was reported during FGDs that some patients provide different names with each clinic visit to obtain medication, while others conceal their border crossings or provide false addresses. Moreover, community leaders often do not report incoming migrants to Thai health authorities or cooperate with disease control teams.

… many times, we couldn’t find the cases because they (patients) lied to our (a malaria civil society organisation) staff that they are residents in Thailand and gave their wrong address. (FMSP, Tak, FGD)

#### Terrain difficulty and telecommunication problems

In FGDs, FMSPs highlighted terrain difficulty as a significant operational barrier that delays RASR activities, particularly during the rainy season. In some areas, FMSPs rely on motorcycles for transportation, incurring additional costs and the need for extra compensation. The delays caused by challenging terrain are further compounded by telecommunication issues. Limited mobile phone networks and internet access disrupt the entire RASR activity cascade. Malaria programme stakeholders noted in FGDs that insufficient internet connectivity hinders not only case reporting to the information system but also the mapping of index cases during case investigations. In some areas, due to restricted network access and difficult terrain, only aggregated monthly surveillance data can be reported, significantly impacting the timeliness and responsiveness of malaria control efforts.

## Acceptability and feasibility of RASR implementation

### Acceptability

All the surveyed malaria programme stakeholders and FMSPs knew the application of 1-3-7 RASR strategy. The majority of malaria programme stakeholders (28/33, 84.9%) and FMSPs (37/41, 90.2%) stated that the 1-3-7 approach was used in primary health care facilities, and advocacy, coordination and information sharing are still needed to enforce hospitals to follow 1-3-7 approach.

Findings from FGDs and IDIs revealed that the RASR strategy, including the 1-3-7 schedule, was generally well-accepted, apart from the 7-day timeframe for completing the focus investigations and response activities. Malaria programme stakeholders and FMSPs agreed that the 1-3-7 strategy helped structure malaria surveillance into a systematic process. With its emphasis on comprehensive reporting and data collection, the strategy facilitated timely detection of malaria outbreaks, streamlining interventions and yielding positive outcomes. Furthermore, the 1-3-7 timeframe motivated lower-performing malaria control teams. Although it is challenging to meet, the clear deadline prompted more active engagement in malaria control activities, as teams worked diligently to meet the 7-day target. As a result, malaria programme stakeholders supported the 1-3-7 strategy.

Similarly, MMPs accepted RASR activities because of their perceived risk of malaria infection and the perceived benefits of the services provided. Some MMPs understood that malaria would not be cured without completing the full course of antimalarial medication, motivating them to cooperate with CI to obtain treatment although antimalarial treatment is provided regardless of patient cooperation in CI. Additionally, MMPs expressed concern about malaria transmission among their children and believed that using long lasting insecticide-treated mosquito nets and performing IRS, the essential interventions in focus response, were effective measures for preventing mosquito bites.

We feel happy when they come to spray. We’re not scared, and we cooperate well with the officers. If we have vegetables at home, we give them to the officers (laughing). (MMP, Tak, FGD)

During IDIs, a malaria programme stakeholder emphasized that the 1-3-7 strategy was not a one-size-fits-all solution. Malaria epidemiology in forest-fringe and border areas is particularly complex, with community awareness and health-seeking behaviours differing from those in other regions. The 1-3-7 approach was perceived as less suitable for these areas, where stakeholders recommended a proactive, rather than reactive, approach to focus response activities, particularly in border regions.

Within region 12 (Yala and Songkhla provinces), we apply the same strategy for all, and it is OK. If we compare (the effectiveness of) the strategy (1-3-7) in the Southern, Northeastern, Northern, and Tak (border area) regions, we may implement different measures/interventions (other than 1-3-7 in Tak). (Malaria programme stakeholder, Songkhla, IDI)

### Feasibility

Findings from IDIs revealed that the feasibility of implementing the 1-3-7 strategy varies significantly depending on geography and malaria incidence. In Songkhla where malaria incidence is low with minimal cross-border cases, the 1-3-7 approach has been effective, allowing RASR activities to be completed on schedule. In contrast, in high-incidence areas such as Tak Province, which borders malaria-endemic Myanmar’s Kayin State, the 1-3-7 strategy faces substantial challenges. Increased malaria cases due to cross-border migration, coupled with logistical barriers such as contacting the cases, have caused delays in RASR activities, limiting the strategy’s feasibility in high-transmission settings. This contrast suggests that while the 1-3-7 approach is feasible and effective in low-incidence areas, its application in high-transmission regions requires adaptation to address unique operational challenges.

The 1-3-7 strategy is suitable for areas with few (malaria cases like Yala (Province). But this won’t work in Tak (Province) because there's a high incidence rate (of malaria). (Malaria programme stakeholder from Songkhla, IDI)

## Discussion

In Thailand, the RASR strategy with a 1-3-7 framework is applied to eliminate malaria, though its perceived feasibility and effectiveness varied by province. Case notification was primarily conducted via telephone calling and LINE group chat, though delays in notifications are common, which affects the timely execution of RASR activities. Policies mandate CI for both indigenous and imported cases, but only about half of reported cases underwent full CI, with variations in RACD practices depending on geographic and epidemiological factors. Although perceived adherence to the 1-3-7 timeframe was generally strong, completing focus response activities within seven days remained challenging, particularly in border areas. Overall, stakeholders recognised the value of the RASR strategy but questioned the feasibility of the 1-3-7 framework in areas with high malaria incidence and unique geographical characteristics.

Delays in reporting malaria cases to district public health offices by hospitals led to late initiation of CI. Improved communication with hospital staff explaining the requirement and importance of the 1-3-7 approach and a policy allowing direct case notification from hospitals to public health centres could expedite CI. Updating the mHealth app, such as streamlined data transmission and accurate location tracking, could further support timely case notification and CI.

Thailand’s policy mandates CI for both indigenous and imported malaria cases [7]. Given Thailand’s proximity to high-burden malaria areas, such as Myanmar, and regions endemic for *Plasmodium knowlesi*, such as Malaysia, this policy is particularly valuable in border areas to prevent the importation and spread of malaria. However, implementing this policy is challenging in border areas, where many migrant cases are reluctant to cooperate in CI—although some MMPs in the FGDs argued that they cooperated in case investigation—due to fears of repercussions related to undocumented migration status. As a result, they often concealed their place of residence, making it difficult to conduct CI. Daily cross-border malaria cases and cases from refugee camps were frequently left uninvestigated, leading to incomplete CI. This gap risks the reintroduction of malaria into malaria eliminated provinces and falling back into a control phase in endemic provinces in Thailand, potentially undermining the country’s progress towards its malaria elimination goal [16, 17].

Participants reported considerable variability in RACD practice in Thailand, in which has also been noted in prior research and national operational plans [7, 8]. This variation reflects differing malaria burdens across regions; areas with higher transmission rates require broader RACD coverage to effectively identify secondary cases [18]. Allocating RDTs and human resources equally to both high and low transmission areas is inefficient, underscoring the need for tailored resource distribution that aligns with local malaria incidence and focus receptivity.

The challenges of RACD implementation are multifaceted and interlinked. Remoteness increases travel time and transportation costs, yet reimbursement for field visits remains fixed per index case within a seven-day period—a limitation stemming from rigid and impractical funding management. In practice, FMSPs often visited remote villages to treat and investigate an index case on day one and conducted RACD on day two. However, if community members were unavailable on the second day, providers may hesitate to return due to inadequate fuel reimbursement, opting instead to screen only the available individuals, often falling short of comprehensive coverage. Additionally, microscopy reimbursement was capped at 50 people, further reducing RACD yield. Adjusting reimbursements to reflect malaria incidence, travel distances, and operational realities would enhance case detection efforts and ensure budget allocations are better aligned with the unique epidemiology and logistical demands of each region. Further, RACD focused more on geographical proximity of the index malaria case (hot spots). It was not carried out in military bases, co-workers, co-travellers and refugee camps, the hot pops, who share the same risk of malaria infection. RACD among hot pops was demonstrated to provide higher case yield in the southern provinces of Thailand [18]. In Cambodia, RACD was found to be more efficacious in low transmission settings when deployed to co-travellers and co-workers compared to when deployed geographically in the hotspots [19]. Since efficiency of the RACD varies between the high and low transmission settings, RACD should target both hot spots and hot pops in accordance with epidemiology of malaria transmission and available resources across the country [18, 20, 21].

Previous studies have shown that the western provinces of Thailand exhibited low adherence to the 1-3-7 timeframe due to higher transmission rates and a greater number of active malaria foci [13]. Completing focus response activities on a case-by-case basis within seven days is challenging in these regions, primarily due to an imbalance between the high volume of cross-border malaria cases and limited human resources. Given these constraints, policymakers should consider alternative response strategies for malaria control in high-incidence areas beyond the standard 1-3-7 approach for malaria elimination. Response activities in these regions should incorporate proactive measures, such as mass screening and treatment, insecticide-treated net distribution, and IRS. Furthermore, a greater emphasis should be placed on comprehensive coverage rather than strict adherence to timelines, with a focus on reaching MMPs on both sides of the border.

Although the 1-3-7 RASR strategy is perceived as effective and acceptable to various malaria stakeholders, it is not a one-size-fit-for-all approach. In urban and low malaria incidence areas, the 1-3-7 RASR strategy remains feasible. However, the retirement and attrition of specialised malaria programme staff has shifted the malaria workload to general health system staff, who may lack sufficient experience in execution of RASR [22]. Continued support from the Department of VBD and expanded training for general health staff will be essential for sustained implementation.

While malaria is recognised as a life-threatening infectious disease by health stakeholders, political commitment in Thailand has remained limited due to historically low prevalence and minimal mortality rates over recent decades. Consequently, budget allocations for malaria elimination have not been prioritised at the policy level. Shrinking domestic and international funding alongside the declining malaria burden limits RASR implementation. Malaria elimination is more resource intensive than malaria control because it requires a more detailed and accurate surveillance system to track and respond to every malaria case [23]. Identifying sustainable funding sources, including domestic funds and international partners, and adjusting budget allocation based on local malaria contexts are crucial [8].

In FGDs, stakeholders in the malaria programme highlighted a notable disconnect between national policy frameworks and local needs. Central policymakers often lack a nuanced understanding of the regional context, leading to inadequate responses to issues raised by local stakeholders. A uniform strategy for RASR has been applied across the country, without considering regional differences in geography, seasonality, and other factors that influence malaria transmission. This blanket approach overlooks critical environmental and climatic variations, particularly between the southern and northern provinces. For instance, Yala Province (neither fully in a control nor elimination phase) was managed under elimination protocols, disregarding the province’s specific epidemiological conditions [13, 24, 25]. Such policies underscore the need for more context-sensitive strategies in malaria policy and programme implementation.

Differences in migration patterns further complicate the malaria response across regions. Western provinces experience significant cross-border migration from Myanmar, while southern provinces primarily manage internal migration. Yet, central authorities have allocated resources uniformly across these regions, disregarding these distinct migration dynamics. Consequently, southern provinces face increased demands, having to test internal migrants both upon entry and exit, which strains resources and leads to testing shortages.

The western province of Tak, which borders Myanmar, presents a unique malaria challenge. Since the onset of Myanmar's political and social crisis in 2021, cross-border movement between Myanmar and Thailand has surged, leading to an increase in malaria cases within Thailand and posing a significant challenge to malaria elimination efforts [5]. Many displaced individuals from Myanmar rely on malaria services in Thailand, necessitating additional resources to meet this growing demand [2]. Furthermore, communities along the border are often less cooperative due to complex issues, including undocumented migration and exploitation within the informal labour market. This may also be due to a lack of awareness of malaria elimination initiatives, including the 1-3-7 strategy, or, in some cases, due to personal interests in maintaining access to inexpensive migrant labour. These behaviours hinder effective malaria surveillance and response efforts, highlighting the need for strategies to increase community awareness and trust.

RASR activities for malaria elimination at the border areas can be challenging because of low access to healthcare services, uncontrolled human and parasite movements, and environmental factors [16, 26]. As highlighted by an IDI participant, a comprehensive situation analysis is essential, given the unique malaria challenges along the Myanmar and Malaysia borders. This analysis should include an epidemiological study of border malaria, geographic characteristics, social determinants such as healthcare infrastructure, entomological surveys, and environmental factors like rainfall and temperature [17]. Such an analysis would provide the foundation for developing strategies to strengthen RASR activities in these border areas.

An additional approach to addressing cross-border malaria RASR is to establish collaborative policies and synergistic action plan with neighbouring countries. Proposed key activities among partnering governments include (1) sharing information on malaria cases and foci, (2) conducting joint RASR activities, (3) resource sharing, (4) funding mobilisation, (5) political advocacy, and (6) infrastructure and capacity building [16] so that all index malaria cases will be notified, investigated and responded to in time. With strong political and technical collaboration, the cross-border RASR activities can be effectively implemented, reducing the risk of malaria reintroduction into Thailand.

Though this study provided valuable insight of how the RASR strategies were being implemented in Tak, Yala and Songkhla provinces, there are some limitations to be acknowledged. Since the staff from malaria clinics and VBD units involved predominantly in the survey, their perspectives on the RASR implementation, adherence to 1-3-7 timeframe and acceptability may not be representative of every malaria stakeholder, particularly the hospital staff. The insights from IDIs and FGDs are also specific to the three study provinces and may not be generalisable to the entire country. Future studies on evaluation of RASR strategies in Thailand should be comprehensive recruiting all related stakeholder categories including those from clinical services provision sector in all provinces where RASR strategies are implemented.

## Conclusions

Thailand’s implementation of the RASR strategy with the 1-3-7 approach has marked progress in malaria control, yet significant barriers persist that threaten the path to elimination. While effective in low-incidence settings, the 1-3-7 framework is fundamentally constrained in high-incidence border regions where limited resources, escalating cross-border migration, accessibility and logistical constraints and complex community dynamics hinder timely and comprehensive responses. Policies must be adapted to the unique challenges of high-transmission areas, with targeted resource allocation, proactive community engagement, and strengthened cross-border cooperation in order to sustain momentum toward malaria elimination (Table 5).

**Table 5:** Summary of issues and policy recommendations

Activities	Issues	Recommendations
Case notification	▪ Delay in case notification by hospitals	▪ Communicate the requirement and importance of 1-3-7 approach to hospital staff ▪ Allow direct case notification from hospitals to public health centres
	▪ Outdated case notification platform	▪ Update mHealth application for streamlined data transmission and accurate location tracking
Case investigation	▪ Mobile and migrant populations are reluctant to cooperate in case investigation* ▪ Daily cross-border cases and cases in refugee camps often left not investigated*	▪ Implement activities to increase community awareness and trust* ▪ Strengthen cross-border political and technical collaborations*
Reactive case detection	▪ Fixed reimbursement amount for every field visit regardless of remoteness* ▪ Inadequate fuel cost reimbursement* ▪ Limited reimbursement for microscopy*	▪ Ensure budget allocations aligned with each region’s unique epidemiology and logistical demands* ▪ Adjust reimbursements to reflect malaria incidence, travel distances and operational realities*
	▪ People who share the same characteristics with the index case (hot populations) are not included in reactive case detection	▪ Expand reactive case detection to hot populations
Focus response	▪ Completing focus response activities within seven days is challenging in border areas due to high volume of cross-border cases and limited human resources*	▪ Prioritise completion of response activities in high burden border areas beyond the standard 1-3-7 timeframe*
Overall reactive surveillance and response strategy	▪ Equal but inefficient allocation of resources to both high and low transmission areas* ▪ Malaria budget allocations not prioritised at the national level	▪ Tailor resources distribution to align with local malaria incidence and focus receptivity* ▪ Identify sustainable funding sources, including domestic funds and international partners
	▪ Challenges underpinned to cross-border malaria: low access to healthcare services, uncontrolled human and parasite movements, and environmental factors*	▪ Perform comprehensive situation analysis along Myanmar and Malaysia borders to provide foundation for developing strategies to strengthen reactive surveillance and response activities in border areas* ▪ Establish collaborative policies and synergistic action plan with neighbouring countries*
	▪ Notable disconnect between national policy frameworks and local needs ▪ Uniform national reactive surveillance and response strategy regardless of regional differences*	▪ Develop and deploy more context-sensitive regional/provincial reactive surveillance and response strategies*

*These issues or recommendations are specific to high transmission border provinces in Thailand

## Supplementary Information

Additional file1 (DOCX 42 kb)

Additional file2 (PDF 376 kb)

Additional file3 (DOCX 67 kb)

## Data Availability

The quantitative and qualitative data generated and analysed in this manuscript will be made available on reasonable request to corresponding authors. Data collection tools are included as Supplementary Material 2 in the submission.

## Abbreviations

API: Annual Parasite Index
CI: Case investigation
FGD: Focus group discussion
FMSP: Frontline malaria service provider
IDI: In-depth interview
IRS: Indoor residual spraying
MMP: Mobile and migrant populations
RACD: Reactive case detection
RASR: Reactive surveillance and response strategy
RDT: Rapid diagnostic test
VBD: Vector-borne diseases

## References

[1] The Mekong Malaria Elimination Programme: Accelerating malaria elimination in the Greater Mekong. In: Bulletin #10. Geneva: World Health Organization; 2022.

[2] World Health Organization: World Malaria Report 2023. Geneva, Switzerland World Health Organization; 2023: 356.

[3] Division of Vector Borne Diseases, Department of Disease Control, Ministry of Public Health, Thailand: Malaria Online: the digital surveillance system for Thailand Malaria elimination. In: UNPSA 2020. 2020.

[4] The Mekong Malaria Elimination Programme. Mekong malaria elimination: epidemiology summary, vol. 17, January–March 2022. Geneva: World Health Organization; 2022.

[5] The Mekong Malaria Elimination Programme. Mekong malaria elimination: epidemiology summary, vol. 47, January–November 2023. Geneva: World Health Organization; 2023.

[6] The Mekong Malaria Elimination Programme. Mekong malaria elimination: epidemiology summary, vol. 59, January–November 2024. Geneva: World Health Organization; 2024.

[7] Department of Disease Control. The ministry of public health, Thailand: national malaria elimination strategy Thailand 2017–2026. Thailand: The Ministry of Public Health; 2016.

[8] Lertpiriyasuwat C, Sudathip P, Kitchakarn S, Areechokchai D, Naowarat S, Shah JA, et al. Implementation and success factors from Thailand’s 1-3-7 surveillance strategy for malaria elimination. Malar J. 2021;20(1):201. 33906648 10.1186/s12936-021-03740-z PMC8076878

[9] Thailand gears up to eliminate malaria by 2024. https://www.who.int/news-room/feature-stories/detail/thailand-gears-up-to-eliminate-malaria-by-2024

[10] von Elm E, Altman DG, Egger M, Pocock SJ, Gøtzsche PC, Vandenbroucke JP. The strengthening the reporting of observational studies in epidemiology (STROBE) statement: guidelines for reporting observational studies. Int J Surg. 2014;12(12):1495–9. 25046131 10.1016/j.ijsu.2014.07.013

[11] O’Brien BC, Harris IB, Beckman TJ, Reed DA, Cook DA. Standards for reporting qualitative research: a synthesis of recommendations. Acad Med. 2014;89(9):1245–51. 24979285 10.1097/ACM.0000000000000388

[12] Thailand Department of Disease Control. Malaria database and mapping in*.* Online: Ministry of Public Health; 2025.

[13] Sudathip P, Naowarat S, Kitchakarn S, Gopinath D, Bisanzio D, Pinyajeerapat N, et al. Assessing Thailand’s 1-3-7 surveillance strategy in accelerating malaria elimination. Malar J. 2022;21(1):222. 35850687 10.1186/s12936-022-04229-z PMC9294779

[14] Green J, Willis K, Hughes E, Small R, Welch N, Gibbs L, et al. Generating best evidence from qualitative research: the role of data analysis. Aust N Z J Public Health. 2007;31(6):545–50. 18081575 10.1111/j.1753-6405.2007.00141.x

[15] Matthew B. Miles AMH, Saldana J. Qualitative data analysis: a methods sourcebook, 4th edn. California, U.S.A: SAGE Publications, Inc.; 2020.

[16] Fambirai T, Chimbari MJ, Ndarukwa P. Global cross-border malaria control collaborative initiatives: a scoping review. Int J Environ Res Public Health. 2022. 10.3390/ijerph191912216. 36231519 10.3390/ijerph191912216 PMC9566216

[17] Li X, Snow RW, Lindblade K, Noor AM, Steketee R, Rabinovich R, et al. Border malaria: defining the problem to address the challenge of malaria elimination. Malar J. 2023;22(1):239. 37605226 10.1186/s12936-023-04675-3 PMC10440889

[18] Kitchakarn S, Naowarat S, Sudathip P, Simpson H, Stelmach R, Suttiwong C, et al. The contribution of active case detection to malaria elimination in Thailand. BMJ Glob Health. 2023. 10.1136/bmjgh-2023-013026. 37940203 10.1136/bmjgh-2023-013026 PMC10632818

[19] Rossi G, Van den Bergh R, Nguon C, Debackere M, Vernaeve L, Khim N, et al. Adapting reactive case detection strategies for falciparum malaria in a low-transmission area in Cambodia. Clin Infect Dis. 2017;66(2):296–8. 10.1093/cid/cix781 29020325

[20] Aidoo EK, Aboagye FT, Botchway FA, Osei-Adjei G, Appiah M, Duku-Takyi R, et al. Reactive case detection strategy for malaria control and elimination: a 12 year systematic review and meta-analysis from 25 malaria-endemic countries. Trop Med Infect Dis. 2023. 10.3390/tropicalmed8030180. 36977181 10.3390/tropicalmed8030180 PMC10058581

[21] Deen J, Mukaka M, von Seidlein L. What is the yield of malaria reactive case detection in the Greater Mekong Sub-region? A review of published data and meta-analysis. Malar J. 2021;20(1):131. 33663484 10.1186/s12936-021-03667-5 PMC7934542

[22] Sudathip P, Kitchakarn S, Thimasarn K, Gopinath D, Naing T, Sajjad O, et al. The evolution of the malaria clinic: the cornerstone of malaria elimination in Thailand. Trop Med Infect Dis. 2019;4(4):143. 31847121 10.3390/tropicalmed4040143 PMC6958345

[23] World Health Organization. A framework for malaria elimination. Geneva: World Health Organization; 2017.

[24] World Health Organization. Malaria 2024 Thailand country profile. World Malaria Report. 2024.

[25] Ammatawiyanon L, Tongkumchum P, Lim A, McNeil D. Modelling malaria in southernmost provinces of Thailand: a two-step process for analysis of highly right-skewed data with a large proportion of zeros. Malar J. 2022;21(1):334. 36380322 10.1186/s12936-022-04363-8 PMC9664774

[26] Wangdi K, Gatton ML, Kelly GC, Clements ACA. Chapter two—cross-border malaria: a major obstacle for malaria elimination. In: Advances in parasitology, vol. 89. In: Rollinson D, Stothard JR, editors. Academic Press; 2015. pp. 79–107. 10.1016/bs.apar.2015.04.002 26003036

