# Supplementary material for: Evaluating Thailand’s malaria reactive surveillance and response strategies for malaria elimination: a mixed-method study
Source: Infect Dis Poverty. 2025 Oct 30;14:109. doi: 10.1186/s40249-025-01382-w (PMC12573910; doi:10.1186/s40249-025-01382-w)
Supplement: Supplementary file 1 — Additional file1 (DOCX 42 kb) [file 40249_2025_1382_MOESM1_ESM.docx]

|  | **Supplementary material 1 – Research checklists**  **Standards for Reporting Qualitative Research (SRQR)*** |  |
| --- | --- | --- |
|  | <http://www.equator-network.org/reporting-guidelines/srqr/> |  |
|  |  | **Page/line no(s).** |
| **Title and abstract** | |  |
|  | **Title** - Concise description of the nature and topic of the study Identifying the study as qualitative or indicating the approach (e.g., ethnography, grounded theory) or data collection methods (e.g., interview, focus group) is recommended | 1 |
|  | **Abstract** - Summary of key elements of the study using the abstract format of the intended publication; typically includes background, purpose, methods, results, and conclusions | 2,3 |
|  |  |  |
| **Introduction** | |  |
|  | **Problem formulation** - Description and significance of the problem/phenomenon studied; review of relevant theory and empirical work; problem statement | 4/56-59, 61-78 |
|  | **Purpose or research questio**n - Purpose of the study and specific objectives or questions | 5/ 80-86 |
|  |  |  |
| **Methods** | |  |
|  | **Qualitative approach and research paradigm** - Qualitative approach (e.g., ethnography, grounded theory, case study, phenomenology, narrative research) and guiding theory if appropriate; identifying the research paradigm (e.g., postpositivist, constructivist/ interpretivist) is also recommended; rationale** | 5/ 91 (post-positivist) |
|  | **Researcher characteristics and reflexivity** - Researchers’ characteristics that may influence the research, including personal attributes, qualifications/experience, relationship with participants, assumptions, and/or presuppositions; potential or actual interaction between researchers’ characteristics and the research questions, approach, methods, results, and/or transferability | 7/ 132 |
|  | **Context** - Setting/site and salient contextual factors; rationale** | 6/106-112  7/ 131-133 |
|  | **Sampling strategy** - How and why research participants, documents, or events were selected; criteria for deciding when no further sampling was necessary (e.g., sampling saturation); rationale** | 7/115-127 |
|  | **Ethical issues pertaining to human subjects** - Documentation of approval by an appropriate ethics review board and participant consent, or explanation for lack thereof; other confidentiality and data security issues | 7/131 |
|  | **Data collection methods** - Types of data collected; details of data collection procedures including (as appropriate) start and stop dates of data collection and analysis, iterative process, triangulation of sources/methods, and modification of procedures in response to evolving study findings; rationale** | 7/130-135, 143 - 146  7-8/148-152 |
|  | **Data collection instruments and technologies** - Description of instruments (e.g., interview guides, questionnaires) and devices (e.g., audio recorders) used for data collection; if/how the instrument(s) changed over the course of the study | 6/103 |
|  | **Units of study** - Number and relevant characteristics of participants, documents, or events included in the study; level of participation (could be reported in results) | 5-6/99–103 |
|  | **Data processing** - Methods for processing data prior to and during analysis, including transcription, data entry, data management and security, verification of data integrity, data coding, and anonymization/de-identification of excerpts | 7/145-148 |
|  | **Data analysis** - Process by which inferences, themes, etc., were identified and developed, including the researchers involved in data analysis; usually references a specific paradigm or approach; rationale** | 7-8/148-152 |
|  | **Techniques to enhance trustworthiness** - Techniques to enhance trustworthiness and credibility of data analysis (e.g., member checking, audit trail, triangulation); rationale** | 8/147-152 |
|  |  |  |
| **Results/findings** | |  |
|  | **Synthesis and interpretation** - Main findings (e.g., interpretations, inferences, and themes); might include development of a theory or model, or integration with prior research or theory | 169-193, 240–242, 296-297, 323-326, 332-335, 360-368, 377-386, 398-470, 480-498, 503, 516-526 |
|  | **Links to empirical data** - Evidence (e.g., quotes, field notes, text excerpts, photographs) to substantiate analytic findings | 8/169-173, 9/175-181, 9/182-185, 9/188-193, 12/232-234, 12/240-242, 13/261-264, 13/265-267, 14/281-282,  14/283-285, 14/296-303,  15/309-313,  16/323-324,  16/327-329, 16/332-335,  16-17/340-344, 17/347-351,  17/352-354, 18/361-368, 18/369-374, 19/382-386,  19/387-389,  20/410-413,  21/426-429,  21/441-442,  22/455-457, 23/491-498,  23/499-500, 24/503-508, 24/509-512,  25/527-529 |
|  |  |  |
| **Discussion** | |  |
|  | **Integration with prior work, implications, transferability, and contribution(s) to the field -** Short summary of main findings; explanation of how findings and conclusions connect to, support, elaborate on, or challenge conclusions of earlier scholarship; discussion of scope of application/generalizability; identification of unique contribution(s) to scholarship in a discipline or field | 25/532-541,  25/543-548, 26/551-553,  26/554-561,  26/570-578,  27/583-584, 27/587-589, 27/595-601, 28/604-619,  28/611-615, 28/621-624, 29/629-631, 29/633-638, 29/640-651, 30/653-656, 30/662-669 |
|  | **Limitations** - Trustworthiness and limitations of findings | 31/676-677 |
|  |  |  |
| **Other** | |  |
|  | **Conflicts of interest** - Potential sources of influence or perceived influence on study conduct and conclusions; how these were managed | Nil |
|  | **Funding** - Sources of funding and other support; role of funders in data collection, interpretation, and reporting | 37/716-723 |
|  |  |  |
|  | *The authors created the SRQR by searching the literature to identify guidelines, reporting standards, and critical appraisal criteria for qualitative research; reviewing the reference lists of retrieved sources; and contacting experts to gain feedback. The SRQR aims to improve the transparency of all aspects of qualitative research by providing clear standards for reporting qualitative research. |  |
|  |  |  |
|  | **The rationale should briefly discuss the justification for choosing that theory, approach, method, or technique rather than other options available, the assumptions and limitations implicit in those choices, and how those choices influence study conclusions and transferability. As appropriate, the rationale for several items might be discussed together. |  |
|  |  |  |
|  | **Reference:** |  |
|  | O'Brien BC, Harris IB, Beckman TJ, Reed DA, Cook DA. **Standards for reporting qualitative research: a synthesis of recommendations.** *Academic Medicine*, Vol. 89, No. 9 / Sept 2014  DOI: 10.1097/ACM.0000000000000388 |  |
|  |  |  |
|  |  |  |

STROBE Statement—checklist of items that should be included in reports of observational studies

|  | Item No. | Recommendation | Page  No. | Relevant text from manuscript |
| --- | --- | --- | --- | --- |
| **Title and abstract** | 1 | (*a*) Indicate the study’s design with a commonly used term in the title or the abstract | 1 | a mixed-method study |
|  |  | (*b*) Provide in the abstract an informative and balanced summary of what was done and what was found | 2, 3 | assess how this 1-3-7 malaria RASR strategy was implemented to understand how it may be optimised to achieve national malaria elimination goals  A cross-sectional mixed-method study was conducted in Tak, Songkhla and Yala provinces of Thailand between January and April 2023  The overall implementation of RASR activities was reported to be timely except for malaria case notification and focus response activities in border areas. Despite good acceptability towards RASR activities, the 1-3-7 strategy is not a one-size-fits-all approach; its feasibility for implementation depends on health system capacity and malaria incidence of the area. |
| Introduction | | | |  |
| Background/ rationale | 2 | Explain the scientific background and rationale for the investigation being reported | 4, 5 | Thailand is a Greater Mekong Subregion country and malaria endemic.  Thailand aims to eliminate malaria in all provinces by 2030.  To effectively implement malaria surveillance and response activities, Thailand adopted 1-3-7 reactive surveillance and response (RASR) strategy, which entails case notification within one day, case investigation (CI) within three days and case investigation survey within seven days following detection of an index malaria.  Since Thailand is accelerating its elimination effort in endemic provinces and preventing reestablishment of malaria in eliminated provinces, it is crucial to effectively implement the RASR strategies so that the surveillance system could capture all malaria cases and prevent onward transmission. |
| Objectives | 3 | State specific objectives, including any prespecified hypotheses | 5 | This paper describes the implementation of RASR activities, adherence to its 1-3-7 timelines and challenges encountered during the implementation and explores the acceptability and feasibility of the RASR strategy among the malaria programme stakeholders, FMSPs and mobile and migrant populations (MMPs) in Tak, Yala and Songkhla provinces of Thailand. |
| Methods | | | |  |
| Study design | 4 | Present key elements of study design early in the paper | 5 | This study is a mixed-method study that employed quantitative cross-sectional surveys, qualitative FGD and semi-structured IDI. |
| Setting | 5 | Describe the setting, locations, and relevant dates, including periods of recruitment, exposure, follow-up, and data collection | 6, 7 | This study was conducted in three provinces of Thailand – Tak, Songkhla and Yala **(Supplementary materials 3: Additional table 2)**.  Between January to April 2023, quantitative and qualitative data collections were conducted confidentially in private locations in the selected provinces. |
| Participants | 6 | (*a*) *Cohort study*—Give the eligibility criteria, and the sources and methods of selection of participants. Describe methods of follow-up  *Case-control study*—Give the eligibility criteria, and the sources and methods of case ascertainment and control selection. Give the rationale for the choice of cases and controls  *Cross-sectional study*—Give the eligibility criteria, and the sources and methods of selection of participants | 6,7 | Participants were recruited purposively based on their roles and experience with implementing malaria control and RASR activities  Subset of FMSPs and malaria programme stakeholders who participated in the surveys were intentionally invited to participate in FGDs for discussion regarding RASR implementation issues based on diversity of their roles in performing RASR activities.  Military and paramilitary staff, and villagers from border areas were purposively selected as MMPs to participate in FGD.  Malaria programme stakeholders who were expert in malaria control, well-experienced with the RASR strategy, and responsible for designing and overviewing RASR policies and strategies were deliberately recruited for the IDI. |
|  |  | (*b*) *Cohort study*—For matched studies, give matching criteria and number of exposed and unexposed  *Case-control study*—For matched studies, give matching criteria and the number of controls per case |  |  |
| Variables | 7 | Clearly define all outcomes, exposures, predictors, potential confounders, and effect modifiers. Give diagnostic criteria, if applicable | 5 | implementation of RASR activities, adherence to its 1-3-7 timelines and challenges encountered during the implementation |
| Data sources/ measurement | 8* | For each variable of interest, give sources of data and details of methods of assessment (measurement). Describe comparability of assessment methods if there is more than one group | - | Surveys |
| Bias | 9 | Describe any efforts to address potential sources of bias | - | The nature of the survey is to provide insight of RASR implementation. Thus, there will not be comparison of outcomes and hence less prone to bias |
| Study size | 10 | Explain how the study size was arrived at | 6 | Sample size was not determined due to limited availability of eligible participants and no intention for inference about the population |

Continued on next page

| Quantitative variables | 11 | Explain how quantitative variables were handled in the analyses. If applicable, describe which groupings were chosen and why | - | The categorical variables were described using frequency and percentage |
| --- | --- | --- | --- | --- |
| Statistical methods | 12 | (*a*) Describe all statistical methods, including those used to control for confounding | - | - |
|  |  | (*b*) Describe any methods used to examine subgroups and interactions | - | - |
|  |  | (*c*) Explain how missing data were addressed | - | Presence of missing values are mentioned in table foot note |
|  |  | (*d*) *Cohort study*—If applicable, explain how loss to follow-up was addressed  *Case-control study*—If applicable, explain how matching of cases and controls was addressed  *Cross-sectional study*—If applicable, describe analytical methods taking account of sampling strategy | - | - |
|  |  | € Describe any sensitivity analyses | - | - |
| Results | | | | |
| Participants | 13* | (a) Report numbers of individuals at each stage of study—eg numbers potentially eligible, examined for eligibility, confirmed eligible, included in the study, completing follow-up, and analysed | 5 | the two surveys were carried out among malaria programme stakeholders (n=33) who were responsible for managing and overseeing the RASR activities and FMSPs (n = 41) who performed the RASR activities |
|  |  | (b) Give reasons for non-participation at each stage | - | - |
|  |  | (c) Consider use of a flow diagram | - | - |
| Descriptive data | 14* | (a) Give characteristics of study participants (eg demographic, clinical, social) and information on exposures and potential confounders | - | Supplementary material 3, additional table 1 |
|  |  | (b) Indicate number of participants with missing data for each variable of interest | - | No |
|  |  | (c) *Cohort study*—Summarise follow-up time (eg, average and total amount) | - | - |
| Outcome data | 15* | *Cohort study*—Report numbers of outcome events or summary measures over time | - | *-* |
|  |  | *Case-control study—*Report numbers in each exposure category, or summary measures of exposure | *-* | *-* |
|  |  | *Cross-sectional study—*Report numbers of outcome events or summary measures | *-* | See the tables |
| Main results | 16 | (*a*) Give unadjusted estimates and, if applicable, confounder-adjusted estimates and their precision (eg, 95% confidence interval). Make clear which confounders were adjusted for and why they were included | - | - |
|  |  | (*b*) Report category boundaries when continuous variables were categorized | - | - |
|  |  | © If relevant, consider translating estimates of relative risk into absolute risk for a meaningful time period | - | - |

Continued on next page

| Other analyses | 17 | Report other analyses done—eg analyses of subgroups and interactions, and sensitivity analyses | - | - |
| --- | --- | --- | --- | --- |
| Discussion | | | | |
| Key results | 18 | Summarise key results with reference to study objectives | 25 | In Thailand, the RASR strategy with a 1-3-7 framework is applied to eliminate malaria, though its perceived feasibility and effectiveness varied by province. Case notification was primarily conducted via telephone calling and LINE group chat, though delayed notifications are common, which affects the timely execution of RASR activities. Policies mandate CI for both indigenous and imported cases, but only about half of reported cases underwent full CI, with variations in RACD practices depending on geographic and epidemiological factors. Although perceived adherence to the 1-3-7 timeframe was generally strong, completing focus response activities within seven days remained challenging, particularly in border areas. Key barriers include limited human resources, budget constraints, community participation issues, difficult terrain, and telecommunication challenges. Stakeholders recognised the value of the RASR strategy but questioned the feasibility of the 1-3-7 framework in areas with high malaria incidence and unique geographical characteristics. |
| Limitations | 19 | Discuss limitations of the study, taking into account sources of potential bias or imprecision. Discuss both direction and magnitude of any potential bias | 30 | there are some limitations to be acknowledged. Since the staff from malaria clinics and VBD units involved predominantly in the survey |
| Interpretation | 20 | Give a cautious overall interpretation of results considering objectives, limitations, multiplicity of analyses, results from similar studies, and other relevant evidence | 31 | Thailand’s implementation of the RASR strategy with the 1-3-7 approach has marked progress in malaria control, yet significant barriers persist that threaten the path to elimination. While effective in low-incidence settings, the 1-3-7 framework is fundamentally constrained in high-incidence border regions where limited resources, escalating cross-border migration, accessibility and logical constraints and complex community dynamics hinder timely and comprehensive responses. Policies must be adapted to the unique challenges of high-transmission areas, with targeted resource allocation, proactive community engagement, and strengthened cross-border cooperation in order to sustain momentum toward malaria elimination. |
| Generalisability | 21 | Discuss the generalisability (external validity) of the study results | 31 | their perspectives on the RASR implementation, adherence to 1-3-7 timeframe and acceptability may not be representative of every malaria stakeholder particularly the hospital staff. |
| Other information | |  | | |
| Funding | 22 | Give the source of funding and the role of the funders for the present study and, if applicable, for the original study on which the present article is based | 34 | This study received funding from National Health and Medical Research Council, Australia (Grant Number: 2017485). Australian Centre for Research Excellence in Malaria Elimination (Grant Number: 1134989) supported funding for FJIF and WHO. The Burnet Institute is grateful for the financial support provided by the Victorian State Government Operational Infrastructure Support grant. It is important to note that the funders did not play any parts in the design of the study, collection and analysis of data, decision to publish, or preparation of the manuscript. |

*Give information separately for cases and controls in case-control studies and, if applicable, for exposed and unexposed groups in cohort and cross-sectional studies.

**Note:** An Explanation and Elaboration article discusses each checklist item and gives methodological background and published examples of transparent reporting. The STROBE checklist is best used in conjunction with this article (freely available on the Web sites of PLoS Medicine at http://www.plosmedicine.org/, Annals of Internal Medicine at http://www.annals.org/, and Epidemiology at http://www.epidem.com/). Information on the STROBE Initiative is available at www.strobe-statement.org.
