# Supplementary material for: Evaluating Thailand’s malaria reactive surveillance and response strategies for malaria elimination: a mixed-method study
Source: Infect Dis Poverty. 2025 Oct 30;14:109. doi: 10.1186/s40249-025-01382-w (PMC12573910; doi:10.1186/s40249-025-01382-w)
Supplement: Supplementary file 3 — Additional file3 (DOCX 67 kb) [file 40249_2025_1382_MOESM3_ESM.docx]

# Supplementary materials 3 – Additional tables

## Additional Table 1. Background characteristics of participants

| **Variable** | **Overall** (*N* = 74) (%) | **Survey 1 (***N* = 33) (%) | **Survey 2** (*N* = 41) (%) | **In-depth interview** (*N* = 3) (%) | **Focus group discussion**  (*N* = 82) (%) |
| --- | --- | --- | --- | --- | --- |
| **Organisation/ workplace*** | | | | | |
| Voluntary health worker/ Youth malaria volunteer | 4 (5.6%) | 0 (0.0%) | 4 (10.3%) |  |  |
| Malaria post / malaria clinic | 20 (27.8%) | 0 (0.0%) | 20 (51.3%) |  | 28(34.1%) |
| Primary health center | 6 (8.3%) | 2 (6.1%) | 4 (10.3%) |  | 3 (3.7%) |
| Vector-borne diseases control center | 27 (37.5%) | 22 (66.7%) | 5 (12.8%) |  | 4 (4.9%) |
| Disease prevention & control office | 2 (2.8%) | 2 (6.1%) | 0 (0.0%) | 3 (100%) | 3 (3.7%) |
| District public health office | 7 (9.7%) | 2 (6.1%) | 5 (12.8%) |  | 1 (1.2%) |
| Provincial public health office | 5 (6.9%) | 5 (15.2%) | 0 (0.0%) |  | 5 (6.1%) |
| Hospital | 1 (1.4%) | 0 (0.0%) | 1 (2.6%) |  | 1 (1.2%) |
| Border guard forces |  |  |  |  | 6 (7.3%) |
| National park |  |  |  |  | 2 (2.4%) |
| Partner/donor |  |  |  |  | 2 (2.4%) |
| Entomology department |  |  |  |  | 1 (1.2%) |
| Mobile and migrant population |  |  |  |  | 11 (13.4%) |
| Not recorded |  |  |  |  | 15 (18.3%) |
| **Age (years)*** |  |  |  |  |  |
| Median (Interquartile Range) | 38.5 (33.0, 48.3) | 36.0 (32.8, 41.0) | 46.0 (33.8, 52.3) |  |  |
| Range | 23–65 | 24–57 | 23–65 |  |  |
| **Gender** |  |  |  |  |  |
| Male | 34 (45.9%) | 12 (36.4%) | 22 (53.7%) |  | 36 (43.9%) |
| Female | 40 (54.1%) | 21 (63.6%) | 19 (46.3%) |  | 15 (18.3%) |
| **Education** |  |  |  |  |  |
| No formal education | 0 (0.0%) | 0 (0.0%) | 0 (0.0%) |  |  |
| Primary school | 1 (1.4%) | 0 (0.0%) | 1 (2.4%) |  |  |
| Secondary school | 4 (5.4%) | 0 (0.0%) | 4 (9.8%) |  |  |
| High school | 18 (24.3%) | 1 (3.0%) | 17 (41.5%) |  |  |
| Degree holder | 45 (60.8%) | 31 (93.9%) | 14 (34.1%) |  |  |
| Others (diploma) | 6 (8.1%) | 1 (3.0%) | 5 (12.2%) |  |  |
| **Duration in current role (years)*** Median (Interquartile Range) | 10.3 (4.0, 17.0) | 10.2 (2.9, 13.2) | 11.6 (4.1, 21.3) |  |  |
| *****Missing values present |  |  |  |  |  |

## Additional Table 2. Study provinces in Thailand

| **Province** | **Longitude** | **Latitude** |
| --- | --- | --- |
| Tak | 98.79329307 | 16.71463492 |
| Songkhla | 100.5456163 | 6.934185309 |
| Yala | 101.2285041 | 6.190745164 |

## Additional Table 3. Village or worksite characteristics of the frontline malaria service providers

| **Variables** | **Frontline malaria service providers** (*N* = 41) |
| --- | --- |
| **Total number of households in village/ worksite catchment area*** Median (Interquartile Range) | 1105 (369, 8125) |
| **Total population in catchment area*** Median (Interquartile Range) | 5200 (2000, 37,813) |
| **Mobile phone signal in village/ worksite *** |  |
| Yes* | 36 (87.8%) |
| Good mobile phone signal* | 29 (70.7%) |
| **Internet access in village/worksite*** |  |
| Yes* | 33 (80.5%) |
| Good internet access to do reporting activities* | 33 (80.5%) |
| *Missing values present |  |

## Additional Table 4. Visiting the index case for case investigation

| **Variable** | **Overall**, *N* = 74 (%) | **Malaria programme stakeholders**, *N* = 33 (%) | **Frontline malaria service providers**, *N* = 41 (%) |
| --- | --- | --- | --- |
| **Visiting index case** |  |  |  |
| No, never | 3 (4.1%) | 2 (6.1%) | 1 (2.4%) |
| Yes, sometimes | 11 (14.9%) | 7 (21.2%) | 4 (9.8%) |
| Yes, always | 60 (81.1%) | 24 (72.7%) | 36 (87.8%) |
| **Appointment with index case*** |  |  |  |
| Telephone the index case | 47 (63.5%) | 23 (69.7%) | 24 (58.5%) |
| No prior communication – go to the index case’s residence to see if they are at home | 9 (12.2%) | 2 (6.1%) | 7 (17.1%) |
| Others* | 15 (20.3%) | 8 (24.2%) | 7 (17.1%) |
| Coordinate with the respective Village Health Worker/Malaria Post/responsible person | 6 (8.1%) | 5 (15.2%) | 1 (2.4%) |
| Issue appointment card | 3 (4.1%) | 1 (3.0%) | 2 (4.9%) |
| Complete case investigation in one day | 3 (4.1%) | 0 (0.0%) | 3 (7.3%) |
| Provide follow-up schedule to patient | 2 (2.7%) | 0 (0.0%) | 2 (4.9%) |
| Go to find patient | 1 (1.4%) | 1 (3.0%) | 0 (0.0%) |
| **When an index case is not at home†** |  |  |  |
| Telephone to schedule an appointment | 62 (83.8%) | 28 (84.9%) | 34 (82.9%) |
| Inform respective Village Health Worker/ Malaria Volunteer to make an appointment with the case | 50 (67.6%) | 25 (75.8%) | 25 (61.0%) |
| Visit the index case a second time | 47 (63.5%) | 22 (66.7%) | 25 (61.0%) |
| Mark as NOT FOUND in the record | 8 (10.8%) | 5 (15.2%) | 3 (7.3%) |
| Mark as IMPORTED in the record | 2 (2.7%) | 2 (6.1%) | 0 (0.0%) |
| No revisiting | 2 (2.7%) | 2 (6.1%) | 0 (0.0%) |
| †Multiple responses  *Missing values present |  |  |  |

This table summarises the practices and activities of malaria programme stakeholders and frontline malaria service providers for case investigation to an index case.

## Additional Table 5. Mapping location and taking travel history of index case for case investigation

| **Variable** | **Overall**, *N* = 74 (%) | **Malaria programme stakeholders**, *N* = 33 (%) | **Frontline malaria service providers**, *N* = 41 (%) |
| --- | --- | --- | --- |
| **Location mapping*** | 60 (81.1%) | 30 (90.9%) | 30 (73.2%) |
| **Location mapping methods†** |  |  |  |
| With the assistance of a software/ application | 37 (50.0%) | 22 (66.7%) | 15 (36.6%) |
| M-Health | 19 (25.7%) | 14 (42.4%) | 5 (12.2%) |
| Geographic Information System/Global Positioning System | 12 (16.2%) | 7 (21.2%) | 5 (12.2%) |
| Google map | 7 (9.5%) | 6 (18.2%) | 1 (2.4%) |
| REVEAL | 3 (4.1%) | 0 (0.0%) | 3 (7.3%) |
| Others: BANGKLAM-DG/ offline map/ a phone application | 3 (4.1%) | 1 (3.0%) | 2 (4.9%) |
| Manual mapping | 17 (23.0%) | 5 (15.2%) | 12 (29.3%) |
| Pinning the index house in an open street map | 9 (12.2%) | 4 (12.1%) | 5 (12.2%) |
| Community map / drawing a map | 8 (10.8%) | 1 (3.0%) | 7 (17.1%) |
| **Travelling history of index case*** | 57 (77.0%) | 30 (90.9%) | 27 (65.9%) |
|  | *n* = 57 (%) | *n* = 30 (%) | *n* = 27 (%) |
| Within district of residence* | 55 (96.5%) | 30 (100.0%) | 24 (88.9%) |
| Outside district of residence* | 48 (84.2%) | 29 (96.7%) | 19 (70.4%) |
| Outside of country* | 40 (70.2%) | 29 (96.7%) | 11 (40.7%) |
| *****Missing values present **†**Multiple responses |  |  |  |

This table summarises the practices and activities of malaria programme stakeholders and frontline malaria service providers for case investigation to an index case.

## Additional Table 6. Criteria and collected information for classification of malaria cases

| **Variables** | **Overall**  *N* = 74 (%) | **Malaria programme stakeholders**  *N* = 33 (%) | **Frontline malaria service providers**  *N* = 41 (%) |
| --- | --- | --- | --- |
| **Participants’ perception of Thailand Department of Vector-Borne Diseases’ definition of imported cases*** | | | |
| Cases detected within the country but from a different province, district, or other administrative unit | 50 (67.6%) | 17 (51.5%) | 33 (80.5%) |
| Cases originating in another country | 4 (5.4%) | 3 (9.1%) | 1 (2.4%) |
| Both of them | 13 (17.6%) | 12 (36.4%) | 1 (2.4%) |
| Others | 3 (4.1%) | 1 (3.0%) | 2 (4.9%) |
| Not using Department’s definition | 2 (2.7%) | 0 (0.0%) | 2 (4.9%) |
| Cases from active foci and residual non-active foci | 1 (1.35%) | 1 (3.03%) | 0 (0.00%) |
| **Collecting and reporting intra-country imported cases*** | 60 (81.1%) | 32 (97.0%) | 28 (68.3%) |
| **Collected information from an index case to determine whether it is an imported or local (indigenous) case†** | | | |
| Village | 50 (67.6%) | 20 (60.6%) | 30 (73.2%) |
| District | 50 (67.6%) | 25 (75.8%) | 25 (61.0%) |
| Province / State | 46 (62.2%) | 23 (69.7%) | 23 (56.1%) |
| Region | 11 (14.9%) | 7 (21.2%) | 4 (9.8%) |
| Country | 28 (37.8%) | 17 (51.5%) | 11 (26.8%) |
| Any of the above | 13 (17.6%) | 8 (24.2%) | 5 (12.2%) |
| Others | 2 (2.7%) | 0 (0.0%) | 2 (4.9%) |
| *****Missing values present  **†**Multiple responses | | | |

## Additional Table 7. Reasons for incomplete case investigation and perceived consequences of failure to do case investigation

| **Variables** | **Overall**  *N* = 74 (%) | **Malaria programme stakeholders**  *N* = 33 (%) | **Frontline malaria service providers**  *N* = 41 (%) |
| --- | --- | --- | --- |
| **Reasons for incomplete case investigation†** | | | |
| The person could not be found | 36 (48.7%) | 17 (51.5%) | 19 (46.3%) |
| Location of the case is not accessible | 21 (28.4%) | 9 (27.3%) | 12 (29.3%) |
| Location of the case is outside of the district of the person responsible for investigation | 14 (18.9%) | 5 (15.2%) | 9 (22.0%) |
| A daily cross-border case | 13 (17.6%) | 10 (30.3%) | 3 (7.3%) |
| An imported case | 10 (13.5%) | 5 (15.2%) | 5 (12.2%) |
| Not enough staff/resources | 3 (4.1%) | 3 (9.1%) | 0 (0.0%) |
| **Consequences of failure to do case investigation*** | | | |
| Source of infection remained unknown / cannot locate the transmission focus | 32 (43.2%) | 15 (45.5%) | 17 (41.5%) |
| Transmission to other areas (cannot control/increase cases/more outbreaks/sudden epidemic) | 31 (41.9%) | 16 (48.5%) | 15 (36.6%) |
| Cannot follow-up for the progress of index case | 3 (4.1%) | 0 (0.0%) | 3 (7.3%) |
| Increasing contact with the malaria at-risk groups and index case | 1 (1.4%) | 0 (0.0%) | 1 (2.4%) |
| **†**Multiple responses *****Missing values present |  |  |  |

## Additional Table 8. Threshold number of index case to trigger reactive case detection and community screening

| **Variable** | **Overall**  *N* = 74 (%) | **Malaria programme stakeholders**  *N* = 33 (%) | **Frontline malaria service providers**  *N* = 41 (%) |
| --- | --- | --- | --- |
| **Applying threshold number of cases detected by passive case detection for triggering reactive case detection*** | | | |
| Yes | 34 (46.0%) | 16 (48.5%) | 18 (43.9%) |
| **Number of index cases required to trigger reactive case detection*** | | | |
| Single confirmed case | 46 (62.2%) | 20 (60.6%) | 26 (63.4%) |
| >1 confirmed cases within a specified radius | 20 (27.0%) | 11 (33.3%) | 9 (22.0%) |
| Other threshold of confirmed cases | 4 (5.4%) | 1 (3.0%) | 3 (7.3%) |
| **Triggering events for reactive case detection** | | | |
| No trigger | 7 (9.5%) | 6 (18.2%) | 1 (2.4%) |
| Local cases only | 43 (58.1%) | 13 (39.4%) | 30 (73.2%) |
| Imported cases only | 7 (9.5%) | 1 (3.0%) | 6 (14.6%) |
| Both local and imported cases | 17 (23.0%) | 13 (39.4%) | 4 (9.8%) |
| **Triggering events for community screening*** | | | |
| No trigger | 0 (0.0%) | 0 (0.0%) | 0 (0.0%) |
| Local cases only | 18 (24.3%) | 10 (30.3%) | 8 (19.5%) |
| Imported cases only | 2 (2.7%) | 0 (0.0%) | 2 (4.9%) |
| Both local and imported cases | 52 (70.3%) | 22 (66.7%) | 30 (73.2%) |
| *****Missing values present **†**Multiple responses | | | |

## Additional Table 9. Criteria to conduct malaria screening around an index case

| **Criterion to screen around an index case** | **Overall** *N* = 74 (%) | **Malaria programme stakeholders** *N* = 33 (%) | **Frontline malaria service providers** *N* = 41 (%) |
| --- | --- | --- | --- |
| **Number of criteria used in screening around an index case** | | | |
| None | 7 (9.5%) | 2 (6.1%) | 5 (12.2%) |
| One criterion | 10 (13.5%) | 3 (9.1%) | 7 (17.1%) |
| Two criteria | 18 (24.3%) | 6 (18.2%) | 12 (29.3%) |
| All three criteria | 39 (52.7%) | 22 (66.7%) | 17 (41.5%) |
| **Criterion used in screening around an index case†** | | | |
| Geographical radius* | 60 (81.1%) | 29 (87.9%) | 31 (75.6%) |
| Number of households* | 54 (73.0%) | 26 (78.8%) | 28 (68.3%) |
| Number of people* | 49 (66.2%) | 26 (78.8%) | 23 (56.1%) |
| **Thresholds** (Median, Interquartile range) | | | |
| Minimum meter of radius screened* | | 1000 (1000, 1000) | 1000 (175, 1000) |
| Minimum number of households screened* | | 10 (10, 10) | 15 (10, 27.5) |
| Minimum number of people screened* | | 50 (50, 50) | 50 (50, 50) |
| *****Missing values present**†**Multiple responses | | | |

## Additional Table 10. Challenges in implementation of reactive surveillance and response activities in Thailand

| **Challenges** | **Overall**  *N* = 74 (%) | **Malaria programme stakeholders**  *N* = 33 (%) | **Frontline malaria service providers**  *N* = 41 (%) |
| --- | --- | --- | --- |
| **Timeliness of case notification*** | | | |
| No barriers for case notification within 1 day | 31 (41.9%) | 7 (21.2%) | 24 (58.5%) |
| **Barriers to timely notification†** | *n* = 43 (%) | *n* = 26 (%) | *n* = 17 (%) |
| Delay in case reporting due to sequential reporting cascade* | 19 (44.2%) | 11 (42.3%) | 8 (47.1%) |
| Mobile network or internet problems* | 17 (39.5%) | 12 (46.2%) | 5 (29.4%) |
| Terrain or transportation difficulty* | 7 (16.3%) | 5 (19.2%) | 2 (11.8%) |
| Patient could not be found, moved out or went back to other areas* after diagnosis | 6 (14.0%) | 4 (15.4%) | 2 (11.8%) |
| **Completeness of case investigation*** | | | |
| No challenges to complete case investigation* | 2 (2.7%) | 2 (6.1%) | 0 (0.0%) |
| **Case investigation challenges†** | *n* = 72 (%) | *n* = 29 (%) | *n* = 41 (%) |
| Patient could not be found* | 17 (23.6%) | 9 (31.0%) | 8 (19.5%) |
| Patient provides incorrect information or not disclose their information* | 14 (19.4%) | 9 (31.0%) | 5 (12.2%) |
| Mobile population / patient moves out* | 9 (12.5%) | 5 (17.2%) | 4 (9.8%) |
| No internet signal / no (phone) signal* | 9 (12.5%) | 3 (10.3%) | 6 (14.6%) |
| Unrest area/terrain or transportation difficulty* | 8 (11.1%) | 6 (20.7%) | 2 (4.9%) |
| Language barrier* | 5 (6.9%) | 3 (10.3%) | 2 (4.9%) |
| Delayed in receiving case report* | 4 (5.6%) | 4 (13.8%) | 0 (0.0%) |
| Resources limitation* | 2 (2.8%) | 2 (6.9%) | 0 (0.0%) |
| **Timeliness of case investigation*** | | | |
| No barriers for case investigation within 3 days* | 41 (55.4%) | 15 (45.5%) | 26 (63.4%) |
| **Barriers to timely case investigation†** | *n* = 33 (%) | *n* = 18 (%) | *n* = 15 (%) |
| Patient could not be found/moved out* | 10 (30.3%) | 8 (44.4%) | 2 (13.3%) |
| Patient provides incorrect information or not disclose their information* | 8 (24.2%) | 5 (27.8%) | 3 (20.0%) |
| Delayed in receiving case report* | 8 (24.2%) | 5 (27.8%) | 3 (20.0%) |
| Terrain or transportation difficulty* | 5 (15.2%) | 0 (0.0%) | 5 (33.3%) |
| Resources limitation* | 2 (6.1%) | 2 (11.1%) | 0 (0.0%) |
| Language barrier* | 2 (6.1%) | 1 (5.6%) | 1 (6.7%) |
| **Community screening** | | | |
| No challenges in community screening* | 19 (25.7%) | 7 (21.2%) | 12 (29.3%) |
| **Community screening challenges†** | *n* = 55 (%) | *n* = 26 (%) | *n* = 29 (%) |
| Minimal/lack of cooperation from community* | 20 (36.4%) | 15 (57.7%) | 5 (17.2%) |
| Unrest area /terrain or transportation difficulty* | 14 (25.5%) | 4 (15.4%) | 10 (34.5%) |
| Mobile populations (especially undocumented)* | 11 (20.0%) | 2 (7.7%) | 9 (31.0%) |
| Resources limitation* | 4 (7.3%) | 2 (7.7%) | 2 (6.9%) |
| Language barrier* | 3 (5.5%) | 1 (3.9%) | 2 (6.9%) |
| Mobile phone network/internet connection problems* | 1 (1.8%) | 1 (3.9%) | 0 (0.0%) |
| **Timeliness of foci investigation*** | | | |
| No barriers for foci investigation within 7 days* | 44 (59.5%) | 17 (51.5%) | 27 (65.9%) |
| **Barriers to timely foci investigation†** | *n* = 30 (%) | *n* = 16 (%) | *n* = 14 (%) |
| Patient provides incorrect information or not disclose their information* | 8 (26.7%) | 4 (25.0%) | 4 (28.6%) |
| Terrain or transportation difficulty* | 7 (23.3%) | 3 (18.8%) | 4 (28.6%) |
| Mobile populations* | 5 (16.7%) | 3 (18.8%) | 2 (14.3%) |
| Resources limitation* | 4 (13.3%) | 4 (25.0%) | 0 (0.0%) |
| Delay in executing 1- & 3- activities* | 3 (10.0%) | 2 (12.5%) | 1 (7.1%) |
| Insecticide spraying machine NOT functioning* | 3 (10.0%) | 3 (18.8%) | 0 (0.0%) |
| Lack of cooperation from community* | 2 (6.7%) | 1 (6.2%) | 1 (7.1%) |
| **Following current reactive surveillance and response guidelines** | | | |
| No barriers to follow current guidelines* | 53 (71.6%) | 24 (72.7%) | 29 (70.7%) |
| **Barriers to following current guidelines†** | *n* = 21 (%) | *n* = 9 (%) | *n* = 12 (%) |
| Resources limitation* | 7 (33.3%) | 6 (66.7%) | 1 (8.3%) |
| Patient could not be found/moved out* | 3 (14.3%) | 0 (0.0%) | 3 (25.0%) |
| Lack of cooperation from community* | 3 (14.3%) | 0 (0.0%) | 3 (25.0%) |
| Terrain or transportation difficulty* | 3 (14.3%) | 1 (11.1%) | 2 (16.7%) |
| Unclear and strict guidelines* | 2 (9.5%) | 2 (22.2%) | 0 (0.0%) |
| **†**Multiple responses *****Missing values present |  |  |  |
